# Effectiveness and safety of polydioxanone thread-embedding acupuncture (TEA) and electroacupuncture (EA) treatment for knee osteoarthritis (KOA) patients with postoperative pain: An assessor-blinded, randomized, controlled pilot trial

**DOI:** 10.1097/MD.0000000000021184

**Published:** 2020-07-24

**Authors:** Ye Ji Lee, Chang-Hyun Han, Ju Hyun Jeon, Eunseok Kim, Jin Youp Kim, Ki Hyun Park, Ae Ran Kim, Eun Jung Lee, Young Il Kim

**Affiliations:** a Department of Acupuncture and Moxibustion Medicine, College of Korean Medicine, Daejeon University; b Clinical Medicine Division, Korea Institute of Oriental Medicine; c Korean Medicine Life Science, University of Science and Technology (UST), Campus of Korea Institute of Oriental Medicine, Daejeon; d Department of Clinical Korean Medicine, Graduate School, Kyung Hee University, Seoul; e Department of Korean Medicine Rehabilitation, College of Korean Medicine, Daejeon University, Daejeon, Republic of Korea.

**Keywords:** knee osteoarthritis, thread-embedding acupuncture, electroacupuncture, randomized controlled trial, study protocol, traditional medicine

## Abstract

**Background:**

Degenerative knee osteoarthritis (KOA) shows an increase in morbidity with improvement in the living conditions and extended lifespans. Treatment for degenerative KOA has been gaining attention since it significantly affects the life of the elderly population and is also associated with increased expenses for medical services and high socioeconomic costs. Treatments for degenerative KOA include nondrug therapy, drug therapy, and surgical treatment. For cases that show little response to conservative treatment but have not involved severe deformation of the knee, procedures such as arthroscopic surgery, autologous chondrocyte implantation, or autologous osteochondral transplantation can be performed. However, effective treatment is required for patients experiencing sustained knee pain after surgery. Although studies confirming the therapeutic effects of acupuncture or thread-embedding acupuncture (TEA) treatment for degenerative KOA have been reported, clinical studies on a combination of TEA and electroacupuncture (EA) in patients complaining of knee pain after arthroscopic surgery, autologous chondrocyte implantation, or autologous osteochondral transplantation have not yet been reported. Therefore, this study aimed to evaluate the effectiveness and safety of this combination treatment in patients with persistent knee pain after arthroscopic surgery, autologous chondrocyte implantation, or autologous osteochondral transplantation.

**Methods/design:**

This study has been designed as a 2-group, parallel, single-center, randomized, controlled, assessor-blinded trial. Thirty-six patients with degenerative KOA who complained of pain even after arthroscopic surgery, autologous chondrocyte implantation, or autologous osteochondral transplantation will be randomized to either the (TEA + EA + Usual care) group or the (Usual care only) group in a 1:1 ratio. The patients in the (TEA + EA + Usual care) group will receive TEA treatment once a week for 4 weeks for a total of 4 sessions and EA twice a week for a total of 8 sessions while continuing usual care. The (Usual care only) group will only receive usual care for 4 weeks. To assess the efficacy of the TEA and EA combination treatment, the visual analogue scale, the Korean version of the Western Ontario and McMaster Universities Osteoarthritis Index, the EuroQol 5-Dimension 5-Level, and the doses of the rescue drug taken will be evaluated at baseline (1W) and weeks 2 (2W), 4 (4W), 6 (6W), and 8 (8W). The primary efficacy endpoint is the mean change in visual analogue scale at week 4 (4W) compared to baseline. Adverse events will be assessed at every visit.

**Discussion:**

This study will provide useful data for evaluating the clinical efficacy and safety of TEA and electroacupuncture combination treatment for improving pain and quality of life after surgery for degenerative KOA.

**Trial registration:**

Clinical Research Information Service of Republic of Korea (CRIS- KCT0004804), March 6, 2020.

## Abstract 2

Supplemental Digital Content is available in the text

## 1 Introduction

Degenerative knee osteoarthritis (KOA) is a disease accompanied by degeneration and wear of the knee joint cartilage and osteochondral defect.^[1]^ Clinical symptoms are knee pain, fatigue, limited range of motion, swelling and tenderness around the joints, friction noises during exercise, and formation of osteophytes.^[2]^ The risk factors for degenerative KOA include gender, increased age, and obesity, and the incidence in women is known to be about three times higher than that in men.^[3]^ Although the prevalence of degenerative KOA slightly differs in studies depending on gender, age, and region, it has been reported to be about 20% to 30% for women and 8% to 15% for men in the middle-aged and elderly population aged over 50 years.^[4]^ The morbidity rate for degenerative KOA has increased with improvements in living conditions and extended lifespans, and the age of onset is also getting younger. This not only significantly affects the life of the elderly population but also increases medical service expenses and socioeconomic costs. Therefore, there is an increasing need for medical knowledge and healthcare services for the middle-aged and elderly patients who suffer from degenerative KOA.^[5,6]^

The treatment methods for degenerative KOA are largely based on nondrug therapy, drug therapy, and surgical treatment. Nondrug therapy involves weight loss, stretching, and muscle-strengthening exercises. If there is no response to nondrug therapy, drug therapy is considered. Drug therapy is classified as topical treatments such as intra-articular steroid injections, hyaluronic acid injections, and capsaicin-based external agents and oral therapies such as nonsteroidal anti-inflammatory drugs, acetaminophen, glucosamine, chondroitin, tramadol, and narcotic analgesics.^[7]^ If pain cannot be resolved with these methods, surgical treatment is considered. Surgical treatments include arthroscopic surgery, autologous chondrocyte implantation, autologous osteochondral transplantation, osteotomy, and arthroplasty. Osteotomy or artificial joint replacement is usually performed in cases of severe joint destruction and deformation, whereas arthroscopic surgery, autologous chondrocyte implantation, and autologous osteochondral transplantation are commonly performed in patients with chronic pain with relatively limited joint destruction.^[8]^

In arthroscopic surgery, an endoscope and the surgical instrument are inserted into the joint through a small incision of less than 1 cm to conduct a diagnostic arthroscopic examination and perform joint irrigation, debridement, or microfracture depending on the patient's condition.^[8]^ The advantages of this procedure include the relatively few complications, short rehabilitation period, and no interference with other subsequent surgeries. But, recent research has shown that the benefits of arthroscopic surgery are lost 1 to 2 years after surgery and that the surgery is not effective in treating osteoarthritis except in cases with acute trauma or symptomatic meniscal injury.^[9,10]^ In autologous chondrocyte implantation, regeneration of cartilage tissue is induced by culturing the collected autologous chondrocytes in vitro for about 3 to 6 weeks and then transplanting them into defective joint cartilage sites.^[8]^ This technique can be applied to areas with relatively large cartilage losses and it causes less damage at the donor site since a very small amount of cartilage tissue is collected. Some research shows therapeutic effects such as pain relief and functional enhancement in over 70% to 90% of cases if the procedure is performed on appropriately selected patients.^[8]^ However, a 15-year long-term follow-up study on patients who received autologous chondrocyte implantation showed 59% failure rate, with limited improvement reported in some of the young patients.^[11]^ Finally, autologous osteochondral transplantation is used for small cartilage defects of about 2 cm^2^ or less. The collected bone cartilages are transplanted into a cartilage defect site.^[8]^ It allows early weight-loading, is inexpensive, and can be performed in a single operation. The progress of this surgery is similar to that of autologous chondrocyte implantation.^[12]^ However, according to a study that followed up patients aged over 40 years for at least 2 years after autologous osteochondral transplantation, 40% of the patients underwent additional artificial joint replacement or another osteochondral transplantation.^[13]^ Thus, there is a need for the safe and effective complementary treatment for patients who have experienced residual pain and discomfort after surgeries but have not deteriorated to the state of absolute knee instability requiring artificial joint replacement or osteotomy.

Thread-embedding acupuncture (TEA) and electroacupuncture (EA) treatments are applied as complementary and alternative therapies for musculoskeletal pain disorders. In TEA, a thread is embedded inside the needle into the acupoint, which can produce a sustained stimulation effect of the embedded thread in addition to stimulation effect of the conventional acupuncture.^[1]^ TEA is used for neck pain,^[14]^ low back pain,^[15]^ shoulder pain,^[16]^ and for sequelae of facial paralysis.^[17]^ A systematic literature review on TEA for degenerative KOA^[18]^ included 3 randomized controlled trials published in China, and all 3 studies showed significant therapeutic effects in the intervention group that received TEA. EA involves the application of electrical stimulation along with acupuncture stimulation, and is widely used in clinical practice to relieve acute or chronic pain.^[1]^ It has been used in the treatment of neck pain,^[19]^ low back pain,^[20]^ and neuropathic pain,^[21]^ and is a method frequently used in the treatment of degenerative KOA. In Korea, EA is recommended as one of the most common treatments for knee pain in clinical practice.^[22]^

Despite the fact that TEA and EA are being used in patients with degenerative KOA in the current clinical setting, there is insufficient clinical evidence to support their use. Furthermore, there have been no reports of clinical trials providing the evidence for a combination of TEA and EA in patients with degenerative KOA who complain of pain and dysfunction even after a certain period of recovery after arthroscopic surgery, autologous chondrocyte implantation, or autologous osteochondral transplantation. Therefore, our researchers planned a preliminary clinical trial for evaluating the efficacy and safety of TEA and EA combination treatment in patients with degenerative KOA who complain of pain even after arthroscopic surgery, autologous chondrocyte implantation, or autologous osteochondral transplantation.

## 2 Methods

### 2.1 Objective

This clinical trial has 2 objectives. First, it aims to evaluate the clinical efficacy of TEA and EA combination treatment by comparing changes in visual analogue scale (VAS) at week 4 between the (TEA + EA + Usual care) group and the (Usual care only) group. Second, it will assess the safety of the TEA and EA combination treatment by examining the adverse events (AEs) after the procedure.

### 2.2 Study design

**Figure 1:**
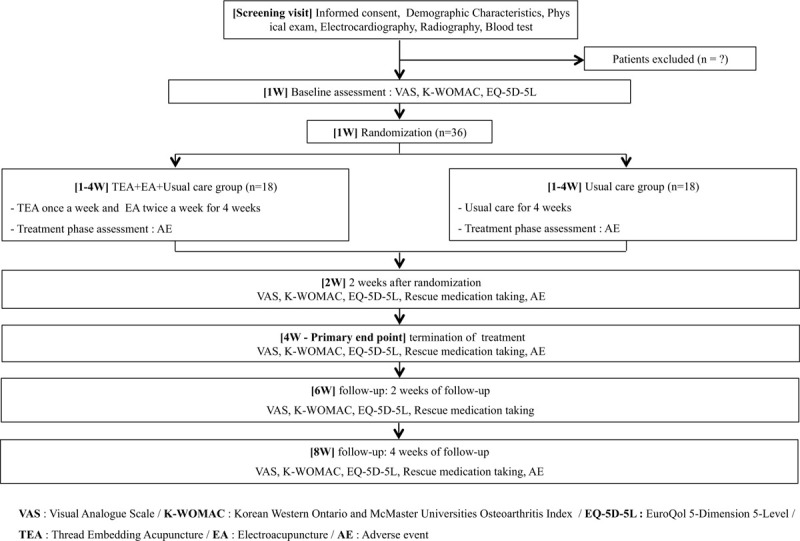
Study flow with outcome assessments.

This study is designed as a 2-group, parallel, single-center, randomized, controlled, assessor-blinded trial. Figure 1 shows the flowchart of this trial. A total of 36 patients will be recruited based on the inclusion/exclusion criteria (Table 1) from subjects who will visit the Daejeon University Daejeon Korean Medicine Hospital between April and December 2020. Subjects will be recruited through the online hospital website and bulletin board, regional newspapers, and advertisement panels on public transportation.

**Table 1:**
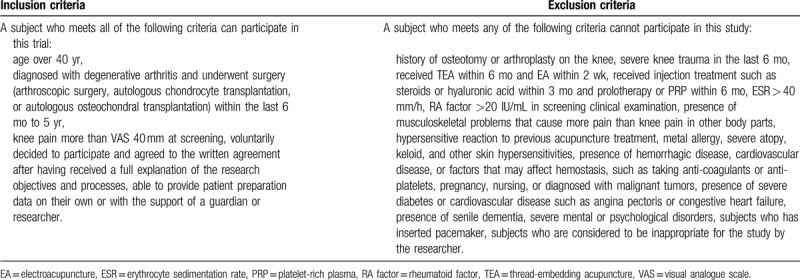
Inclusion and exclusion criteria.

Subjects voluntarily consent to participating in the clinical trial and sign the written consent form. A series of surveys and tests will be performed to determine eligibility, which include a demographic information survey, interview on medical history, questionnaire assessments, blood tests, electrocardiogram examinations, knee radiographic examinations, and urine human chorionic gonadotropin tests for women of childbearing potential. Once eligibility has been determined, the subjects will be notified of their availability to participate, and eligible subjects will be randomized to the (TEA + EA + Usual care) group and (Usual care only) group in a 1:1 ratio. The (TEA + EA + Usual care) group will receive TEA treatment once a week for 4 weeks for a total of 4 sessions and EA twice a week for a total of 8 sessions while continuing usual care. The (Usual care only) group will only receive usual care for 4 weeks. During the 8-week study period, there will be 3 visit assessments (weeks 2, 4, and 8) and 1 nonvisit assessment (week 6).

### 2.3 Sample size

This is a preliminary pilot study designed to assess the feasibility of the study and collect information on the sample size calculation for the entire study. Therefore, the sample size has been assumed to be 14 per group and 28 in total, considering the minimum range that allows efficacy assessment and the number of subjects that can be recruited during the planned study period, rather than being based on statistical calculations. Considering the dropout rate of 20%, the actual recruitment number has been determined to be 18 per group and 36 in total.

### 2.4 Randomization and blinding

Randomization should be performed to ensure that treatment assignment is not biased and it is not exposed to the subjects as well as the investigators. A statistician unrelated to conducting and assessment of the clinical trial will randomly assign 18 subjects to each group using the statistical program SAS Version 9.4 (SAS institute. Inc, Cary, NC) with the same probability for each individual. The independent statistician will keep the randomization table to make sure that it is not disclosed.

Since the participants will be divided into the (TEA + EA + Usual care) group and (Usual care only) group, blinding of the practitioners and subjects will be difficult due to the significant differences in the procedures performed in each group. However, the assessors will be blinded to control the bias as much as possible. Efficacy assessments for subjects will be conducted by investigators who have not performed the intervention and the randomization. The Assessors simply ask questions on evaluation items and case report forms (CRFs), and they are blinded to the treatment the subjects are receiving.

### 2.5 Interventions

#### 2.5.1 Thread-embedding acupuncture combined with electroacupuncture

Table 2^[1,18,23–26]^ and Table 3^[1,22,27,28]^ summarize of the details of TEA and EA treatments. The subjects in the (TEA + EA + Usual care) group will receive the combined treatment consisting of TEA once a week and EA twice a week for 4 weeks while continuing usual care. TEA and EA treatments will be performed by a licensed Korean Medicine doctor with at least 3 years of clinical experience in TEA. The procedure site will be thoroughly disinfected using 78% alcohol cotton before and after the procedure to prevent infection.

**Table 2:**
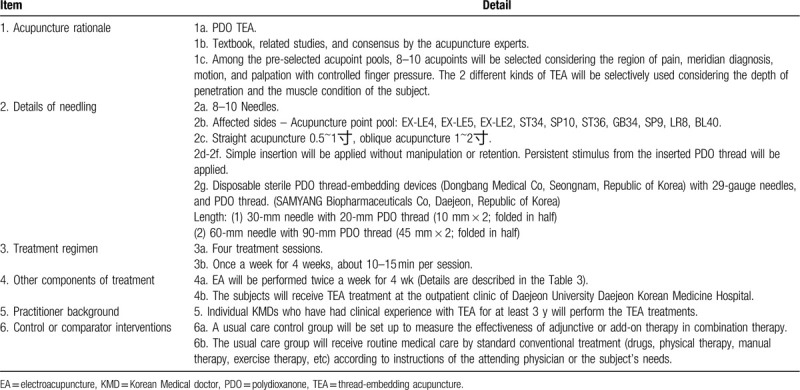
TEA treatment details based on standards for reporting interventions in clinical trials of acupuncture (STRICTA) checklist.

**Table 3:**
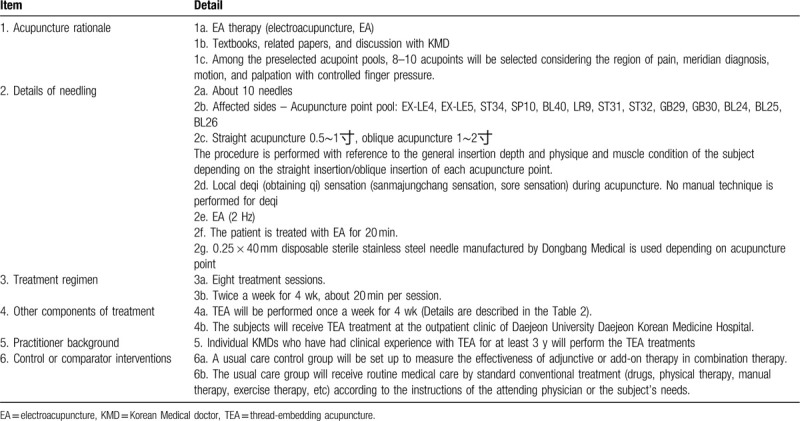
EA treatment details based on standards for reporting interventions in clinical trials of acupuncture (STRICTA) checklist.

For TEA, a 29-gauge 30-mm needle or a 29-gauge 60-mm needle with polydioxanone threads manufactured by Dongbang Medical Co will be used. The appropriate length will be determined based on the depth and direction of the insertion at each acupoint and the subject's physique and muscle conditions. The needles will be inserted after properly exposing the procedure site by having the patient bend the knee at 45° in a sitting position. The practitioner will select 8 to 10 acupoints from the acupoint pool of EX-LE4, ST35 = EX-LE5, EX-LE2, ST34, SP10, ST36, GB34, SP9, LR8, and BL40 based on the region of pain, meridian diagnosis, motion, and palpation with controlled finger pressure.

For EA, gamma-sterilized disposable needles (0.25 × 40 mm, Dongbang Medical Co, Korea) will be used. After the subject is placed in the lateral position and the procedure site is properly exposed, acupuncture will be performed. The needles will be inserted into approximately 10 acupoints from the acupoint pool of EX-LE4, ST35 = EX-LE5, ST34, SP10, BL40, LR9, ST31, ST32, GB29, GB30, BL24, BL25, and BL26, including the fixed 6 acupoints ST34, SP10, BL40, LR9, BL24, and BL25. Electrical stimulation will be given at 2 Hz for 20 minutes on (ST34-SP10), (BL40-LR9), and (BL24-BL25).

#### 2.5.2 Usual care

The participants in the (Usual care only) group will receive conventional usual care alone for 4 weeks. Usual care in this study is defined as routine medical care by standard conventional treatment. The participants will receive usual care such as drug therapy, physical therapy, manipulation, and exercise therapy for knee pain relief depending on their individual needs. After the end of the clinical trial, up to 2 sessions of TEA compensatory treatment will be provided for the subjects of the (Usual care only) group who wish to receive it.

#### 2.5.3 Cointerventions

All subjects will be allowed to receive usual care for knee pain relief and instructed to report the details and the frequency of usual care received during the study period to the investigators in charge. However, other Korean medical treatments for knee pain relief such as acupuncture (eg, acupuncture, EA, pharmacopuncture, TEA), moxibustion, herbal medicine, cupping, etc are not permitted, and invasive treatments (injections and surgical treatment) on the knee are also prohibited. The investigators will record significant drug/nondrug therapy in the CRF.

In this study, acetaminophen with a maximum dose of 3000 mg (6 T/day) or less per day will be provided as the rescue drug, and subjects will be instructed to take and report it only when the pain is severe and unbearable during the clinical study period. Assessments will be performed without taking the rescue drug on the day of assessment for weeks 2, 4, 6, and 8, and the rescue drug can be taken if necessary after the assessment has been completed.

Other concomitant medications (including treatment drugs for other diseases or AEs) that are believed to not have a significant impact on the interpretation of the results of this clinical trial will be allowed at the investigator's discretion. If a drug is taken arbitrarily by the subject without prior notice or the decision of the investigator and this drug is believed to have significant effect on the assessments of this clinical trial, the subject will be dropped out.

### 2.6 Outcome measures

Assessments of clinical variables will be performed at baseline (1W) and weeks 2 (2W), 4 (4W), 6 (6W), and 8 (8W). Table 4 shows the assessment schedule.

**Table 4:**
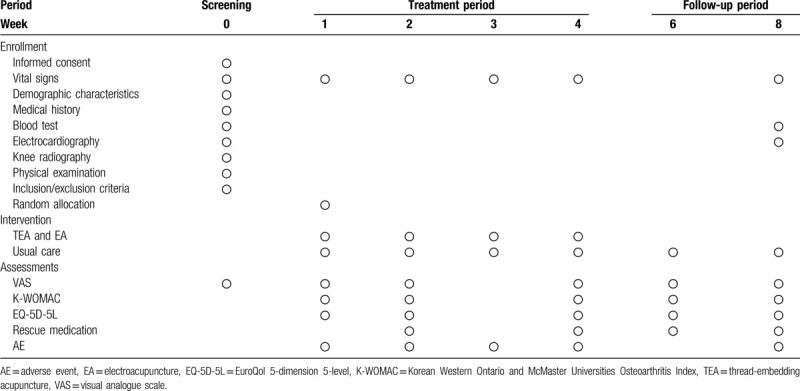
Schedule for the treatment and the outcome measurements.

The primary endpoint is the mean change in VAS score at 4W. VAS is one of the methods to grasp the patient's subjective pain scale, and the patient marks the pain on a 100-mm straight line. In general pain assessment, the left end of the line indicates no pain while the right end indicates the maximum pain imaginable. In this study, the severity of the symptoms will be indicated by a straight vertical line drawn on the scale for the knee pain felt within the last 48 hours, which will be used by the assessor for measurement with a ruler.^[29]^

The secondary endpoints include the mean change in VAS at 2W, 6W, and 8W, the mean change in the individual scores of three subscales (pain/stiffness/physical functioning) and the total score for the Korean Western Ontario and McMaster Universities Osteoarthritis Index (K-WOMAC) at 2W, 4W, 6W, and 8W, the mean change in EuroQol 5-Dimension 5-Level scores at 2W, 4W, 6W, and 8W, and the mean dose of rescue drug taken assessed at 2W, 4W, 6W, and 8W.

The K-WOMAC is a tool for measurement of pain in osteoarthritis patients and is one of the most widely used indicators of overall knee joint function. It can comprehensively evaluate knee joint pain, disability, and the limitations in certain functions. The K-WOMAC consists of 24 questions in total with three subscales, including 5 questions on pain, 2 questions on stiffness, and 17 questions on other physical functions of difficulties in performing related daily activities. This tool is a self-assessment tool completed by the patient that assesses the condition in the past 48 hours, and takes about 3 minutes to complete. Each question is answered on a 5-point Likert scale (0 = none, 1 = mild, 2 = moderate, 3 = severe, and 4 = extreme). The range of scores is 0 to 20 for pain, 0 to 8 for stiffness, and 0 to 68 for physical function. The sum of the scores for these scales is the total K-WOMAC score, which ranges from 0 to 96. High scores in the assessment indicate deterioration in symptoms, limited activities, and bad health.^[30,31]^

The EuroQol 5-Dimension 5-Level is a tool for assessing the quality of life that was newly created to improve the sensitivity and the ceiling effect of EuroQol 5-Dimension 3-Level (EQ-5D-3L). Subjects check off the most appropriate sentence for their health condition under 5 areas, including mobility, self-care, usual activities, pain/discomfort, and anxiety/depression. Each item checked off is indicated by a number from 1 to 5 (1 = no problems, 2 = slight problems, 3 = moderate problems, 4 = severe problems, and 5 = extreme problems), and the weighted combination of these 5 numbers is expressed as an index between 0 and 1, where 0 indicates death and 1 indicates full health.^[32]^

The rescue drug necessary for knee pain management will be provided to the subjects during the study period and the dose taken will be assessed. Evaluating the dosage of rescue drugs provides information on the analgesic effects of the intervention, as effective pain control in the intervention group will reduce the required dose of the rescue drug. The subjects will receive sufficient rescue drug and a dosing diary during the study period. Instructions will be given to fill out the dosing diary every day and to bring it as well as the remaining rescue drug at the time of visits, so as to check the dosage of rescue drug.^[33]^

### 2.7 Adverse events

In this study, AEs refer to all undesirable medical findings that appear after the start of the clinical trial. The medical conditions and diseases that existed before the trial are considered as AEs only if they worsen after starting the treatment. Subjects will be instructed to voluntarily report AEs as they appear, and the investigator will perform AE assessments at every visit based on the results of vital signs, interviews, and other examinations. Regardless of the relationship to the intervention, all identified AEs will be recorded in the CRF. In cases serious adverse events (SAEs) occur during the study period, they will be reported to the Institutional Review Board (IRB) and the monitoring agent within 24 hours of awareness and the clinical trial for the corresponding intervention will be stopped. In addition, follow-up reports will be submitted with additional safety information until AEs terminate.

### 2.8 Statistical analysis

Analysis is carried out using the program SAS Version 9.4 (SAS institute. Inc, Cary, NC). All statistical analyses are two-tailed test, and the significance level will be set to 5%. For missing values, multiple imputation is used.

The full analysis set and the per-protocol set will be used in the analysis of data obtained from the subjects. The full analysis set refers to minimizing subjects excluded from analysis and analyzing the set of subjects excluded only for justifiable reasons. The per-protocol set refers to the analysis of the subject group with no protocol violations who have completed at least 75% of the participation level (at least 3 TEA and at least 6 EA in the intervention group) and all measurements for endpoints indicated in the protocol.

Descriptive statistics are presented for the demographic characteristics and basic clinical data such as gender, age, medical history, and medication history by intervention group. For continuous variables, comparison by each group will be analyzed by an independent *t* test or Wilcoxon rank sum test after presenting the means and standard deviations, along with the 95% confidence intervals, if necessary. For categorical variables, analysis will be carried out using the Chi-squared test or Fisher Exact test after presenting the frequency and percentiles.

The primary efficacy endpoint is the mean change in VAS at week 4 compared to baseline, and will be analyzed using an analysis of covariance with each group as the fixed factor and the VAS before intervention and age as the covariates. If there is a clinical difference between groups in demographic and preintervention characteristics, it will be corrected by adding it as a covariate if needed. The analysis for secondary endpoints will be carried out using the same method as for the primary efficacy endpoints. In addition, to analyze the differences in measurements before and after the intervention within each group, a paired *t* test or Wilcoxon signed-rank test will be used for the primary and secondary efficacy endpoints. To test for the difference in trends by visit, repeated-measures analysis of variance will be performed. For multiple comparison correction, Dunnett procedure will be used.

Safety assessments will be mainly performed by analyzing the incidence of AEs and SAEs that are suspected to be related to the intervention by the investigator. AEs will be collected through symptom reports from subjects and observations by the investigator. The frequency of AEs related and unrelated to the intervention will be recorded and presented as descriptive statistics.

### 2.9 Withdrawal and dropout

The study completion of all subjects participating in the clinical trial will be recorded by the investigators. Reasons will be recorded in case the procedure is discontinued or if a subject is dropped out. The study will be discontinued for subjects in case a violation of the inclusion/exclusion criteria is discovered during the study, in case of protocol violations, or in case continuation of the clinical trial is difficult due to the occurrence of an AE or a SAE.

### 2.10 Ethics and monitoring

This study has been approved by the Institutional Review Board of the Daejeon University Deajeon Korean Medicine Hospital (DJDSKH-20-BM-01) and has been registered in the clinical trial information service (KCT0004804), one of the primary registries for the World Health Organization (WHO) International Clinical Trial Registration Platform. All matters in this clinical trial will be conducted in accordance with the Declaration of Helsinki.

Subjects may express their wish to give up participation in the clinical trial at any time if they no longer wish to participate. Monitoring of the clinical trial will be performed by a monitor agent designated by the Korean Institute of Oriental Medicine. The monitor agent will check the progress of the clinical trial through visits or telephone calls and verify the original subject records and procedure records. After study completion, the originals of the data obtained through CRFs and other procedures during the course of the study will be stored separately in accordance with the IRB regulations of the Daejeon Korean Medicine Hospital, Daejeon University. Upon completion of the study, an independent researcher will conduct statistical analysis of the data. Records that can identify the subjects will remain confidential when the results of the clinical trial are published.

## 3 Discussion

This study has been designed to evaluate the clinical efficacy and safety of a combination of TEA and EA in patients with degenerative KOA with postoperative pain. Many studies have reported results that assessed the efficacy of TEA^[23,25]^ or EA^[34,35]^ in patients with degenerative KOA, but there have been no reports assessing combined treatment with TEA and EA in patients having pain and dysfunction despite undergoing arthroscopic surgery, autologous chondrocyte implantation, or autologous osteochondral transplantation after being diagnosed with degenerative KOA. Therefore, the present study aims to assess the safety and efficacy of the TEA and EA combination treatment in patients with degenerative KOA who complain of pain and dysfunction even after a certain period of recovery after arthroscopic surgery, autologous chondrocyte implantation, or autologous osteochondral transplantation.

Eligibility to participate in the clinical trial will be determined by reviewing the inclusion and exclusion criteria of this study in patients with degenerative KOA who have persistent pain after undergoing arthroscopic surgery, autologous chondrocyte implantation, or autologous osteochondral transplantation in the past 6 months to 5 years. Medical history interview, electrocardiogram examination, blood test, and knee radiographic examinations will be performed during screening. SAEs that may result from the combination treatment of TEA and EA will be prevented by excluding subjects with significant clinical findings in these tests.

However, this pilot study has the disadvantages with a small sample size and short period of intervention. Moreover, due to significant differences in the interventions performed in the 2 groups, practitioner or subject blinding will not be possible, resulting in possible nonspecific effects such as placebo effect or treatment expectation. Nevertheless, this study has the design which reflects the general clinical setting such as usual care and the rescue drug, and is the first clinical trial on the effects of TEA and EA combination treatment on pain and quality of life of patients. This study will provide useful data in assessing the clinical efficacy and safety of TEA and EA combination treatment for improving pain and quality of life in patients with degenerative KOA.

## 4 Trial status

This trial is currently in the recruitment phase, and recruitment began in April 2020. The trial is expected to be completed by November 30, 2020, so results should be published by 2021.

## 5 Data sharing statement

The data from this trial will be accessible by contacting the corresponding author. The trial findings will be disseminated through open-access journals and at national and international conferences.

## 6 Ethics approval and consent to participate

This study protocol was prepared according to the Standard Protocol Items: Recommendations for Intervention Trials (see Supplemental Digital Content (Appendix S1)) and was approved by the IRBs of the hospitals to which the participating research centers belong: Daejeon University Deajeon Korean Medicine Hospital (DJDSKH-20-BM-01). The study will be performed in accordance with the approved protocol, and written informed consent will be obtained from every participant.

## 7 Trial registration

This trial was registered with the Clinical Research Information Service of South Korea (KCT0004804) on 6 March 2020.

## Author contributions

**Conceptualization:** Ye Ji Lee, Young Il Kim.

**Funding acquisition:** Chang-Hyun Han.

**Investigation:** Ye Ji Lee.

**Methodology:** Eunseok Kim, Jin Youp Kim, Eun Jung Lee.

**Software:** Ki Hyun Park.

**Supervision:** Young Il Kim, Chang-Hyun Han.

**Validation:** Ae Ran Kim.

**Writing – original draft:** Ye Ji Lee.

**Writing – review & editing:** Ju Hyun Jeon.

## Supplementary Material

Supplemental Digital Content
